# NanoViromics: long-read sequencing of dsRNA for plant virus and viroid rapid detection

**DOI:** 10.3389/fmicb.2023.1192781

**Published:** 2023-06-21

**Authors:** Vahid J. Javaran, Abdonaser Poursalavati, Pierre Lemoyne, Dave T. Ste-Croix, Peter Moffett, Mamadou L. Fall

**Affiliations:** ^1^Saint-Jean-sur-Richelieu Research and Development Centre, Agriculture and Agri-Food Canada, Saint-Jean-sur-Richelieu, QC, Canada; ^2^Centre SÈVE, Département de Biologie, Université de Sherbrooke, Sherbrooke, QC, Canada; ^3^Département de phytologie, Faculté des Sciences de l’Agriculture et de l’Alimentation, Université Laval, Québec, QC, Canada

**Keywords:** dsRNA, total RNAs, grapevine viruses, virus detection, viroid, nanopore sequencing, Illumina Miseq sequencing, plant pathology

## Abstract

There is a global need for identifying viral pathogens, as well as for providing certified clean plant materials, in order to limit the spread of viral diseases. A key component of management programs for viral-like diseases is having a diagnostic tool that is quick, reliable, inexpensive, and easy to use. We have developed and validated a dsRNA-based nanopore sequencing protocol as a reliable method for detecting viruses and viroids in grapevines. We compared our method, which we term direct-cDNA sequencing from dsRNA (dsRNAcD), to direct RNA sequencing from rRNA-depleted total RNA (rdTotalRNA), and found that it provided more viral reads from infected samples. Indeed, dsRNAcD was able to detect all of the viruses and viroids detected using Illumina MiSeq sequencing (dsRNA-MiSeq). Furthermore, dsRNAcD sequencing was also able to detect low-abundance viruses that rdTotalRNA sequencing failed to detect. Additionally, rdTotalRNA sequencing resulted in a false-positive viroid identification due to the misannotation of a host-driven read. Two taxonomic classification workflows, DIAMOND & MEGAN (DIA & MEG) and Centrifuge & Recentrifuge (Cent & Rec), were also evaluated for quick and accurate read classification. Although the results from both workflows were similar, we identified pros and cons for both workflows. Our study shows that dsRNAcD sequencing and the proposed data analysis workflows are suitable for consistent detection of viruses and viroids, particularly in grapevines where mixed viral infections are common.

## Introduction

Grape cultivation is of major economic importance in many countries, especially for wine production. In 2021, more than 7.3 million ha of various varieties were planted in vineyards around the world (International Organization of Vine and Wine, 2022). Nevertheless, an increase in the number of outbreaks of viral and viroid diseases, which have a negative impact on grapevine growth and yields, fruit quality, and vineyard lifespan, represent a serious threat to the grapevine industry (Martelli, 2017). A total of 95 viruses from 18 families and 38 genera, along with seven viroids from one family (*Pospiviroidae*) and four genera, have been identified in infected grapevines (Fuchs, 2020; Javaran et al., 2021; Read et al., 2022; Roy and Fuchs, 2022). In the absence of effective chemical compounds for controlling viral diseases, managing grapevine viruses is challenging (Armijo et al., 2016). Viruses are not only able to adapt to a variety of environmental situations and new hosts, but can evolve rapidly through mutation, genetic drift, and genetic recombination (Sanjuán and Domingo-Calap, 2021). A number of other factors, including long-term continuous monoculture, climate change, the global trade in plant materials, and the expanding geographical ranges of insect vectors, have also led to an increase in viral diseases (Elena et al., 2011, 2014; Lefeuvre et al., 2019; He et al., 2022). Consequently, growers need to identify viruses as early as possible in order to take timely action and implement the necessary sanitary measures (Wallingford et al., 2015; Fuchs, 2020; Javaran et al., 2021).

Although a number of advanced and traditional diagnostic methods are available for detecting grapevine viruses—including immunological techniques (Borges et al., 2020), nucleic acid amplification (Rowhani et al., 2017), microarrays (Engel et al., 2010), and hyperspectral imaging (Bendel et al., 2020; Nguyen et al., 2021)—the inability of these methods to simultaneously detect all known viruses as well as novel and unknown viruses is still one of their major limitations. The introduction of second-generation sequencing (SGS) has resulted in the detection and identification of many novel and known grapevine viruses, including grapevine Syrah virus 1 (GSV1), grapevine vein clearing virus (GVCV), grapevine pinot gris virus (GPGV), grapevine virus F (GVF), grapevine red blotch virus (GRBV), grapevine roditis leaf discoloration-associated virus (GRLDaV), grapevine virus N (GVN) and grapevine virus O (GVO) (Al Rwahnih et al., 2009; Calvi, 2011; Zhang et al., 2011; Maliogka et al., 2015; Fall et al., 2020; Read et al., 2022). Although SGS has been used to detect and discover known and unknown viruses and its great potential as a diagnostic tool has been recognized, its limitations make it slower to use in diagnostic laboratories than other methods. These limitations include laborious and expensive library preparation and data management techniques, expensive sequencing equipment, and the need for sophisticated technical expertise in order to analyze the data (Pop and Salzberg, 2008; Buermans and den Dunnen, 2014; Olmos et al., 2018; Maclot et al., 2020). Furthermore, in routine diagnostic laboratories, a small number of samples may need to be sequenced, and using SGS would not be economically viable (Pecman et al., 2022). Some of these limitations have been addressed by the introduction of third-generation sequencing (e.g., nanopore sequencing technology) (Mikheyev and Tin, 2014; van Dijk et al., 2018; Javaran et al., 2021).

A number of features of nanopore sequencing, such as the small size of the sequencer (MinION), ease of library preparation, low sequencing cost, possibility of long-read sequencing, and the rapid sequencing process, make it an excellent tool for the surveillance of viruses and other pathogens (Phannareth et al., 2020; Javaran et al., 2021; Sun et al., 2022). Various nanopore RNA and DNA sequencing kits have been used in plant virus detection, and this sequencing technology has shown potential in diagnostic applications. Since RNA viruses make up a majority of plant viruses, cDNA and native RNA-based kits (such as direct RNA sequencing, direct cDNA sequencing, and cDNA-PCR sequencing) are frequently used (Javaran et al., 2021; Sun et al., 2022). Because RNA viruses do not usually have poly(A) tails, library preparation requires a number of modifications when nanopore sequencing kits employing poly(T) adapters are used. Two options are available for sequencing poly(A)-tailed and non-poly(A)-tailed viruses: the use of random hexamer primers in cDNA synthesis, which requires the use of a cDNA sequencing kit, and the addition of several adenine nucleotides to the 3′ end of RNA with *Escherichia coli* poly(A) polymerase (Sun et al., 2022). In addition to RNA viruses, nanopore sequencing technology has also been used to detect a number of DNA viruses (both single and double stranded). For instance, a nanopore sequencing rapid barcoding kit, which can be used in the field, was able to detect the African and East African cassava mosaic viruses (Boykin et al., 2019).

Although different nucleic acid types (DNA or RNA) have been used in detecting plant viruses with nanopore sequencing (Bronzato Badial et al., 2018; Filloux et al., 2018; Boykin et al., 2019; Chalupowicz et al., 2019; Fellers et al., 2019; Naito et al., 2019; Della Bartola et al., 2020; Leiva Ana et al., 2020; Ben Chehida et al., 2021; Pecman et al., 2022), the use of double-stranded RNA (dsRNA), which is generated during the virus replication process, for nanopore sequencing has only been reported twice, and has involved single virus infections: new isolates of jasmine virus C (Amoia et al., 2022) and cucumber Bulgarian latent virus (Dong et al., 2022). Total RNA is generally used in plant virus detection (Liefting et al., 2021; Pecman et al., 2022), although it has a number of limitations. Most reads from total RNA sequencing derive from host transcripts, such as rRNA and mRNA. It is essential to remove host plant RNAs, particularly rRNA, before preparing total RNA libraries for virus detection. The poly(A)-based rRNA removal procedure does not work when capturing plant viruses without poly(A) tails, and alternative methods can are expensive, especially when using commercial kits (Thompson et al., 2020) such as the QIAseq FastSelect Plant Kit. This kit was utilized in this study because it was effective in depleting rRNA from grapevine samples, but its high cost makes it less cost-effective for large-scale diagnostics. A good alternative for detecting plant viruses is the use of dsRNA, (Al Rwahnih et al., 2015; Marais et al., 2018; Ma et al., 2019; Gaafar and Ziebell, 2020). Although negative-sense single-stranded RNA viruses (-ssRNA) were not initially proven to produce dsRNAs during replication (Weber et al., 2006), recent viromic studies have shown that these viruses generate dsRNAs in small amounts (Elbeaino et al., 2018; Samarfard et al., 2020; von Bargen et al., 2020). In our previous research, using dsRNA allowed us to detect not only RNA viruses and viroids, but also a DNA virus, the grapevine red blotch-associated virus (GRBV) (Fall et al., 2020; Xu et al., 2021; Lussier-Lepine et al., 2023). Therefore, dsRNA is a suitable starting material for the detection of viruses regardless of their genomic materials.

The aim of this study was to introduce a simple nanopore dsRNA (dsRNAcD) sequencing protocol, for utilization in both detection and evolutionary studies. We describe a step-by-step protocol that can be used in the diagnostic testing of infected grapevine samples with the Oxford Nanopore Technologies (ONT) MinION sequencing device. Grapevines were selected since this challenging plant is a host for multiple viruses, resulting in many mixed virus infections, as well as containing substances like polyphenols and polysaccharides, which can interfere with reverse transcription and enzymatic reactions during library preparation process. The dsRNA extraction and library preparation protocols for grapevines were optimized by taking into account the sequencing cost per sample. In these experiments, direct RNA and direct cDNA sequencing kits were used for library preparation, and the performance of each kit in detecting viruses was tested under various experimental conditions. In addition, the results were weighed against those from Illumina sequencing in order to compare the performance of the two sequencing technologies in detecting viruses. Moreover, a cost-effectiveness analysis was performed to determine when this technology should be used. Finally, two different bioinformatics workflows, which can be used for diagnostic purposes or evolutionary studies, were evaluated for suitability with our sequencing approach. Overall, dsRNAcD sequencing has considerable potential in plant virus and viroid detection and the genomic characterization of mixed infections. In addition, it can greatly reduce sequencing costs; multiple samples can be sequenced on the same flow cell simultaneously, which could lead to substantial cost savings compared to SGS.

## Materials and methods

### Plant materials

A total of 24 asymptomatic and symptomatic grapevine samples (a combination of leaves and petioles) were collected from a vineyard at Agriculture and Agri-Food Canada’s experimental farm in Frelighsburg, Quebec (latitude 45°03′12′′ N, longitude 72°51′42′′ W) (Supplementary File S1). Samples were collected from grapevine plants (*Vitis vinifera* ‘Vidal blanc’) over the course of July and September 2019 and placed in sterile 50-mL centrifuge tubes and transferred to cold storage at 20°C. The leaves were washed with distilled water, roughly crushed, and homogenized in liquid-nitrogen-cooled 50-mL conical centrifuge tubes with eight stainless-steel balls (8 mm) using a 600 MiniG^®^ Tissue Homogenizer and Cell Lyser (SPEX^®^ SamplePrep). Then, the powdered leaves (1.5–2 g) were transferred to sterile 50-mL centrifuge tubes and stored at −80°C to await nucleic acid extraction.

### dsRNA extraction

A modified version of the dsRNA extraction protocols developed by Fall et al. (2020) and Kesanakurti et al. (2016) was used to extract dsRNA from 24 different grapevine samples. In brief, 12 mL of extraction buffer (200 mM Tris [pH 8.3], 10 mM EDTA, 300 mM lithium chloride, 55 mM lithium dodecyl sulfate, 25 mM deoxycholic acid, 2% PVP-40000, 1% Nonidet P-40, and 1% 2-mercaptoethanol) were added to 1.5 g of homogenized leaf samples. In addition, a positive control, *Phaseolus vulgaris* cv. Black Turtle Soup (BTS), known to be infected by *Phaseolus vulgaris* endornavirus 1 (PvEV1) and *Phaseolus vulgaris* endornavirus 2 (PvEV2) (Kesanakurti et al., 2016; Fall et al., 2020), was added at a final concentration of 1% (w/w) in each sample to assess the efficiency of the dsRNA extraction protocol. After 40 min of shaking at 300 rpm, the tubes were centrifuged at 1000 x g for 1 min at 10°C to remove the bubbles and debris. The supernatant was transferred to a new 50-ml tube, 12 mL of potassium acetate buffer (5.8 M) was immediately added, and the tubes were centrifuged at 14,000 x g for 15 min at 10°C. After the supernatant was transferred to another clean 50-ml centrifuge tube, 16 mL of 100% isopropanol was added, and the tubes were stored at −20°C for 20 min. Centrifugation was performed at 11,000 x g for 16 min at 4°C, the supernatant was discarded, and the pellet was dissolved in STE-18 buffer (10 mM Tris [pH 8.0], 100 mM NaCl, 1 mM EDTA [pH 8.0], and 18% ethanol). Next, 300 mg of Sigmacell Cellulose Type 101, dissolved in 2 mL of STE-18, was added to the solution. The tubes were shaken at 300 rpm for 15 min at room temperature and then centrifuged at 14,000 x g for 5 min, and the supernatant was discarded. To eliminate single-stranded RNAs and DNAs, two washing steps were performed using STE-18, the first with 40 mL and the second with 20 mL. The supernatant was removed by centrifuging at 14,000 x g for 5 min at 20°C between washing steps. Finally, to elute the extracted dsRNA, 6 mL of 1XSTE (10 mM Tris [pH 8.0], 1 mM EDTA [pH 8.0], and 100 mM NaCl) was added to the cellulose pellet and the solution was stirred for 15 min on the shaker. After centrifugation at 14,000 x g for 8 min at 20°C, the supernatant was transferred to a new 50-ml centrifuge tube, and 3 M sodium acetate (pH 5.2) and ethanol were used to precipitate the dsRNAs. The detailed protocol can be found on the protocols.io website.^1^

### Extraction of total RNA

Three different samples were randomly selected from the 24 samples collected and the total RNAs were extracted from 100 mg of leaf material using the RNeasy Plant Mini Kit (Qiagen, Canada) in accordance with the MacKenzie et al. (1997) protocol. Quantitative and qualitative measurements of the total RNAs were performed using a NanoDrop 2000c spectrophotometer (Thermo Scientific, Canada) and a Qubit 4 FLuorometer (Life Technologies, Canada).

### Preparation of dsRNAcD sequencing libraries

To ensure the complete removal of ssRNAs and DNAs, dsRNAs were digested with DNase I and RNase T1. Digestion was halted by adding 50 mM of EDTA and heating at 65°C for 10 min. The double-stranded RNA was denatured at 99°C for 5 min in the presence of 2 μL of 60 μm random primers, 1 μL of 10 mM deoxyribonucleotide triphosphate (dNTP), and 6 μL of water. Then, the tubes were immediately placed in ice water and a master mix (4 μl First-strand cDNA Synthesis Buffer, 1 μL RNaseOUT or RNasin^®^ Ribonuclease inhibitor [40 u/μl], and 1 μL [200 units] of Maxima H minus) was added. The reverse transcription step was performed for 90 min at 55°C. One unit of Ribonuclease H was then used to hydrolyze the DNA–RNA duplex. The second strand of cDNA was synthesized by adding Klenow DNA Polymerase I and *E. coli* DNA Ligase I. Agencourt AMPure XP magnetic beads (Beckman-Coulter) were used to clean up the two-stranded cDNAs. The detailed protocol can be found on the protocols.io website.^2^

**Figure 1:**
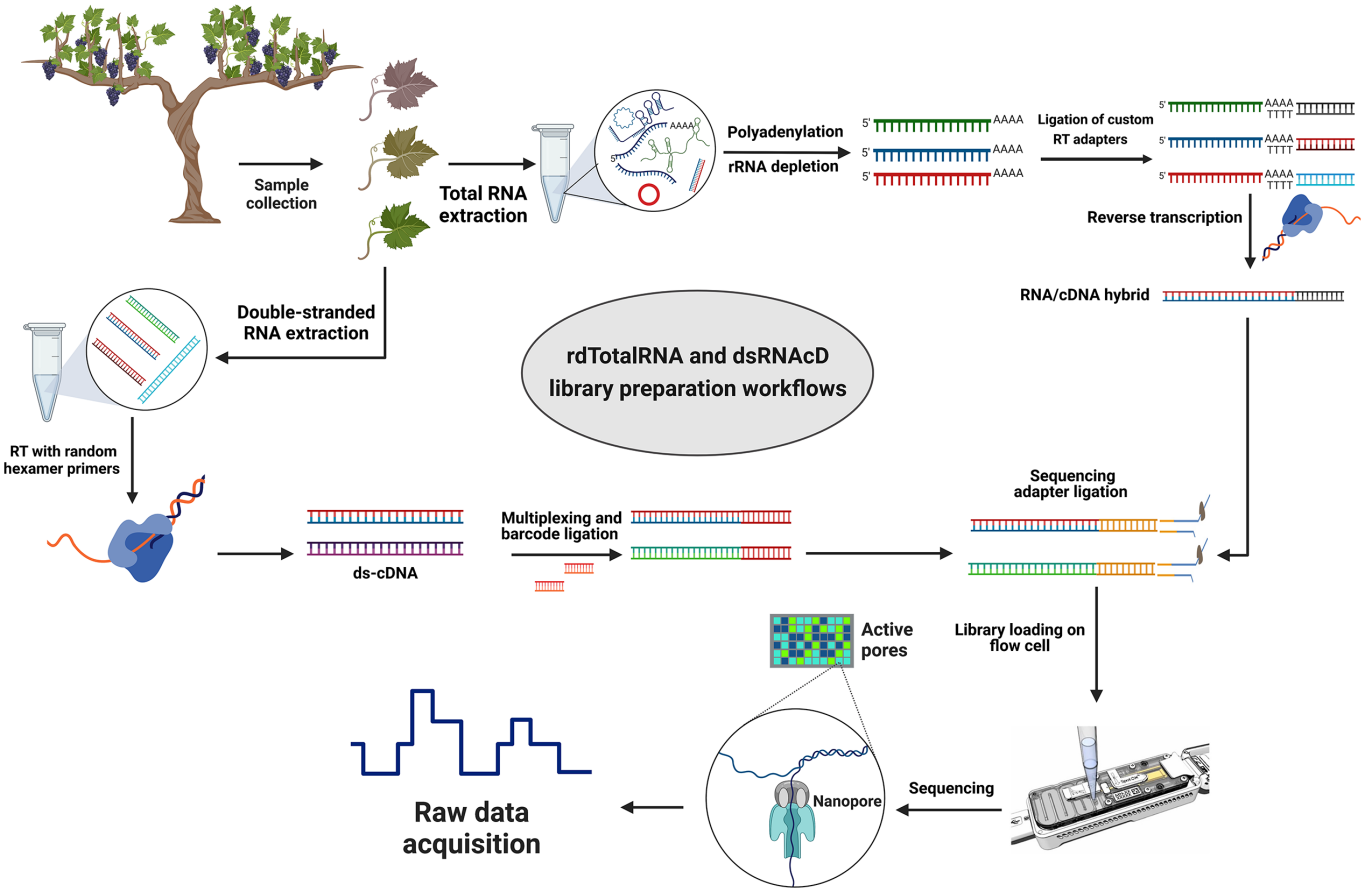
Library preparation workflows for grapevine virus and viroid detection. Direct RNA sequencing (top): Initially, rRNAs were depleted from total RNA and then polyadenylated. Next, different custom reverse transcription adapters were ligated to polyadenylated transcripts, and after reverse transcription (RT), the sequencing adapter was ligated, and a pooled library was loaded on a R9.4.1 flow cell. Direct cDNA sequencing (left-down): Following dsRNA extraction, double-stranded cDNA was synthesized using random primers followed by Klenow polymerase and *E. coli* DNA ligase to create second strand cDNA. A commercial barcode was ligated to each sample after double-stranded cDNAs were end-prepared. The pooled library was made from 23 different barcoded samples, and after ligating the sequencing adapter, the pooled library was primed and loaded on another R9.4.1 flow cell. (The figure was designed by BioRender.com).

Using the direct cDNA sequencing kit (SQK-DCS109, ONT) and its associated protocols, two libraries of cDNA samples were generated. Initially, 24 cDNA samples from various infected grapevines were pooled to prepare a library (referred to as the pooled library) using the direct cDNA sequencing protocol (DCS_9090_v109_revO_14Aug2019) without multiplexing barcodes. Using the direct cDNA sequencing kit and native barcoding kits EXP-NBD104 and EXP-NBD114, the second library (referred to as the multiplexed library) was prepared for 23 different cDNA samples according to the manufacturer’s recommendations (Figure 1); one sample from the 24 samples collected failed. Since cDNA synthesis was performed using random primers, the library preparation process was started from the “end-prep” step of the aforementioned protocol using the NEBNext Ultra II End Repair/dA-tailing Module (New England Biolabs [NEB]). Blunt/TA Ligase Master Mix (NEB) was then used to ligate the sequencing adapter (AMX) to the pooled library. The multiplexed library was constructed by ligating a native barcode to each sample using Blunt/TA Ligase Master Mix. In order to pool the barcoded samples in equal proportions, the quantity of each sample was measured with a Qubit dsDNA HS Assay Kit and a Qubit 4.0 fluorometer. As a final step, the NEBNext Quick Ligation Module (NEB E6056) was used to ligate the sequencing adapter (AMII) to the multiplexed library. Following each enzymatic step of the protocol, AMPure XP magnetic beads were used to purify the samples.

### Preparation of direct RNA sequencing libraries

Since the direct RNA sequencing kit (SQK-RNA002) from ONT is optimized for poly(A)-tailed transcripts, several modifications were made to the direct RNA sequencing library preparation protocol to capture both poly(A)-tailed and non-poly(A)-tailed viral RNAs. After DNase I digestion and the removal of rRNAs from the total RNA using the QIAseq FastSelect -rRNA Plant Kit probe (QIAGEN), several adenine bases were tailed at the 3′ end of the remaining RNAs according to the protocol in Liefting et al. (2021). The samples were then multiplexed using three pre-annealed RT adapters obtained from Integrated DNA Technologies (IDT), using the DeePlexiCon method (Supplementary File S2). In brief, each custom RT adapter was ligated to 500 ng of rRNA-depleted and poly(A)-tailed RNA samples using T4 DNA Ligase (NEB M0202L), which was followed by reverse transcription using SuperScript III Reverse Transcriptase (Thermo Fisher Scientific). The cDNA/RNA hybrid complexes resulting from reverse transcription were purified using Agencourt RNAClean XP beads. The Qubit dsDNA HS Assay Kit was used to measure each sample’s concentration, and 65 ng of reverse-transcribed RNA was taken from each sample to pool the samples in equal concentrations. The RNA sequencing adapter (RMX) was ligated to the RNA-cDNA hybrid complex using T4 DNA Ligase (NEB M0202L) and subsequently purified with Agencourt RNAClean XP beads (Beckman-Coulter) at a 1X ratio according to the Direct RNA Sequencing protocol (Figure 1).

### Priming and loading the R9 flow cell, sequencing, demultiplexing, and base-calling

Three different nanopore sequencing libraries (Table 1) were loaded on three MinION R9.4.1. (FLO-MIN106D) flow cells by using the Flow Cell Priming Kit (EXP FLP002) according to the manufacturer’s instructions. The sequencing step was carried out on a MinION Mk1B device, and the sequencing conditions were set up using MinKNOW software (v.21.11.8). Following 24 h of sequencing and raw data acquisition, the raw data were base-called and demultiplexed with Guppy software (v6.0.6). A score of seven was used as the minimum for quality, and reads below this score were removed. In the direct RNA sequencing experiment, raw data were base-called using Guppy software (v6.0.6), and demultiplexing was carried out using the DeePlexiCon software tool (Smith et al., 2020) according to the developer’s instruction.

**Table 1:** A description of the samples, sequencing kits, and library preparation information used in this study.

	Number of samples	Sequencing kit	Material	Multiplexing
Library 1	24	Direct cDNA sequencing (SQK-DCS109)	dsRNA	None
Library 2	23	Direct cDNA sequencing (SQK-DCS109)	dsRNA	Native Barcoding Expansion kits^a^ (EXP-NBD104 and EXP-NBD114)
Library 3	3	Direct RNA sequencing (SQK-RNA002)	Total RNA	Custom barcodes^b^
Library 4	23^c^	Illumina Nextera XT DNA library prep kit	dsRNA	Unique dual indexes

a Native Barcoding Expansion kits contain 24 unique barcodes for multiplexing and sequencing different samples simultaneously on one flow cell.

b Custom barcodes are pre-annealed RT adapters which were designed based on the DeePlexiCon method for multiplexing and sequencing different RNA samples simultaneously on one flow cell.

c The dsRNA materials used for MiSeq sequencing were the same as those used for dsRNAcD sequencing.

### Quality control and preprocessing of datasets

The raw sequencing data from the three nanopore sequencing libraries were evaluated for quality and descriptive statistics using NanoPlot (version 1.33.0) (De Coster et al., 2018). To trim each dataset individually, separate quality plots from the head and tail regions of reads were depicted by NanoQC (v0.9.4). The head and tail of each read were then trimmed using NanoFilt (v2.8.0) (De Coster et al., 2018). Host sequence contamination was removed by aligning the reads against the grapevine genome (GCF_000003745.3_12X) using Minimap2 software (v2.17-r941) (Li, 2021) for the nanopore sequencing datasets and bowtie2 (Langmead and Salzberg, 2012) for the MiSeq datasets, and host-related reads were excluded using SAMtools (v1.6) (Danecek et al., 2021) to increase data analysis speed and accuracy. Clustering, error correction, and polishing of the trimmed and filtered datasets were performed using the Rattel toolbox (v1.0) (De la Rubia et al., 2022) in accordance with the developer’s instructions for nanopore sequencing datasets. In the case of direct RNA sequencing, Rattle (De la Rubia et al., 2022) was run using the “*-y rna*” option.

### Illumina library preparation and sequencing

We synthesized 23 cDNA samples using the same dsRNA materials that were used for dsRNAcD library preparation, according to the cDNA synthesis procedure described in the section on dsRNAcD library preparation. An Illumina Nextera XT DNA Library Preparation Kit (catalog number FC-131-1,096) was used for this process, using 1 ng of double-stranded cDNA as input. Paired-end sequencing was carried out using the MiSeq Reagent Nano Kit v2 in combination with an Illumina MiSeq sequencer as described in Fall et al. (2020).

## Analysis of nanopore sequencing data

### Strategy 1: Virus and viroid detection with the Centrifuge-Recentrifuge (Cent&rec) workflow

**Figure 2:**
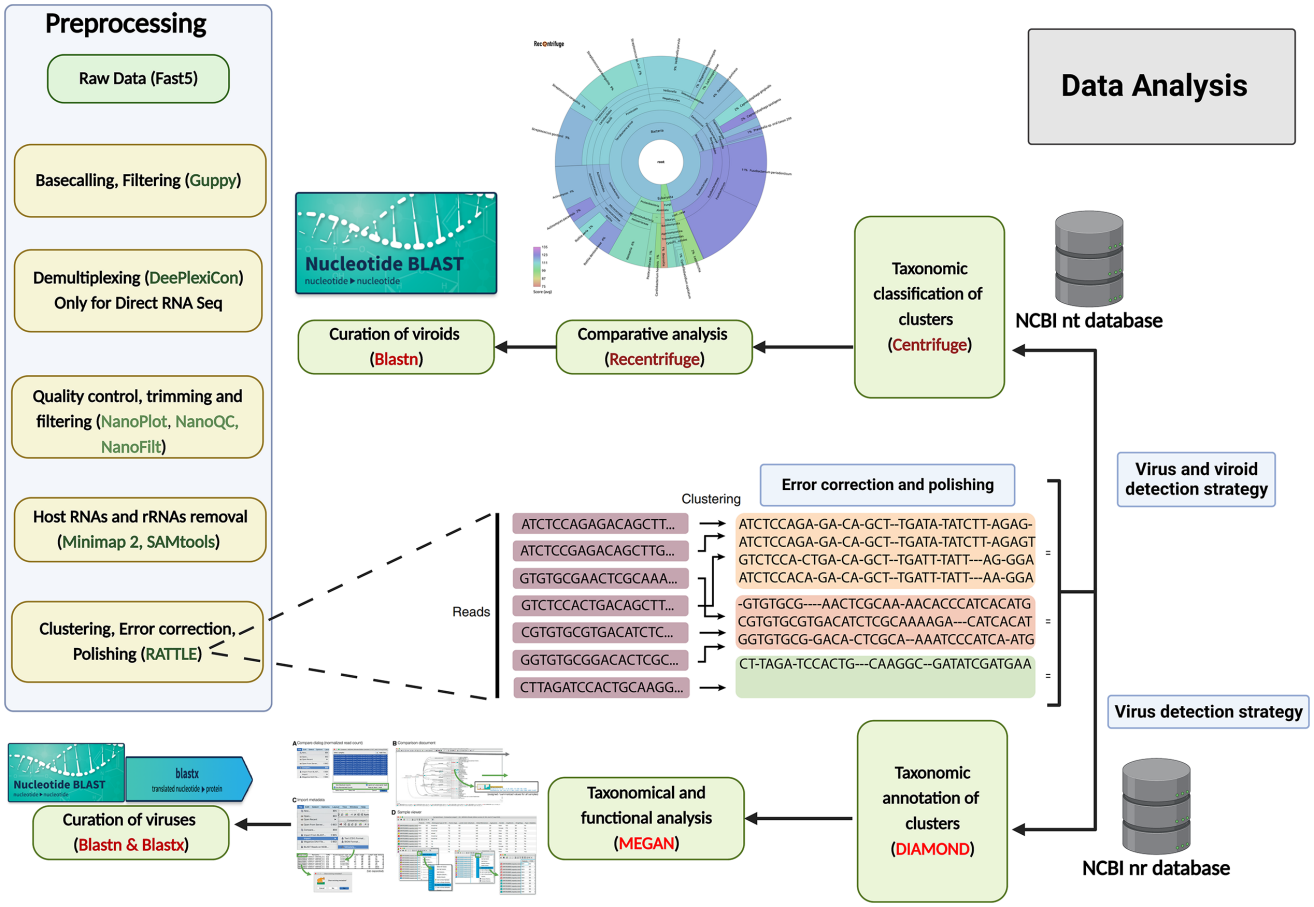
Data analysis workflows for grapevine virus and viroid detection through nanopore sequencing. Preprocessing of raw data (left): The raw data were acquired through MinKNOW software, and then base-calling, filtering based on the quality score (<7), demultiplexing, trimming, the removal of host reads and clustering were performed, followed by error correction and polishing, using various software and software packages. Centrifuge and Recentrifuge strategy (Upper right): Preprocessed reads were taxonomically classified using Centrifuge, followed by a comprehensive analysis by Recentrifuge, which provided a visualization. DIAMOND+MEGAN strategy (Lower right): In addition, the same reads from the previous step were aligned against annotated protein sequences (NCBI-nr) using DIAMOND, and subsequently, MEGAN 6 was used to bin the sequences based on their taxonomy and function profiles. To further curate the taxonomic results, BLASTn and BLASTx were also run.

After quality control, read trimming, cleaning, clustering, error correction, and polishing (the preprocessing step in Figure 2), the long reads were taxonomically classified with a Centrifuge classifier (version 1.0.4) using an in-house workflow with a customized Centrifuge indexing database (CID) (Figure 2) (Kim et al., 2016). The CID was constructed using a local database (created in December 2021), which included GenBank, RefSeq, the TPA and PDB genomes, virus gene and transcript sequence data, as well as the *Homo sapiens* GRCh38p13 genome assembly, bacteria and archaea genome assemblies, and the genome assemblies in ViroidDB. First, the Centrifuge classifier performed taxonomic assignment using default values. Then, the Recentrifuge software (version 1.9.1) (Martí, 2019) was used to perform a comparative analysis of the classification results and to produce interactive HTML reports using the *-y* 50 option (Figure 2). To further curate the taxonomic results, BLASTn (Basic Local Alignment Search Tool) and BLASTx (Sayers et al., 2021) were also run this portion of the protocol.

### Strategy 2: Virus detection with the DIAMOND-MEGAN (DIA&MEG) workflow

The corrected and clustered reads were aligned against a database of annotated protein sequences (NCBI-nr) in order to carry out the taxonomic and functional binning of the sequences according to the procedure developed by Bağcı et al. (2021). However, some minor modifications were involved: instead of processing long-read datasets through a de-novo assembly step as described by Bağcı et al. (2021), we used the DIAMOND protein aligner (Buchfink et al., 2021) to align the corrected reads directly against NCBI-nr (thus eliminating the de-novo assembly step), and then MEGAN 6 to complete the taxonomic and functional binning steps (Gautam et al., 2022). One modification was made to the meganization step, with the “weighted” parameter selected instead of the “longReads” parameter. MEGAN 6 was also used to perform an interactive analysis of the results and to extract the comparative bar charts and taxonomic trees (Figure 2). To further curate the taxonomic results, BLASTn and BLASTx (Sayers et al., 2021) were run.

### Illumina MiSeq data analysis

The short reads obtained from MiSeq sequencing were analyzed in two ways: (1) the Lazypipe pipeline (Plyusnin et al., 2020) was used for the de-novo assembly of the short reads, followed by taxonomic profiling by the Centrifuge classifier and then a comparative analysis by Recentrifuge; (2) the DIAMOND-MEGAN workflow described above was used to align the short reads against NCBI-nr and produce comparative bar charts and taxonomic trees.

### Viral genome coverage

In order to measure genome coverage for detected viruses, the workflow began with removing host reads from short and long raw reads by Minimap2 v2.26 (Li, 2021), BWA-MEM2 v2.2 (Vasimuddin et al., 2019), and SAMtools v1.17 (Li et al., 2009). The viral and viroid genomes were downloaded from the Viral RefSeq database in order to create a local database. Next, filtered reads were mapped against each virus and viroid in the local database using Minimap2 (for long read mapping) and BWA-MEM2 (for short read mapping). We used SAMtools and BEDTools v2.31.0 (Quinlan and Hall, 2010) for format conversions, sorting, indexing, and coverage calculations. For each reference genome, key metrics such as coverage and depth of coverage were calculated and used for further analysis. In order to visualize the processed data in the form of a heat map, Python libraries including Pandas v2.0.1 (McKinney, 2010), Seaborn v0.12.2 (Waskom, 2021), Matplotlib v3.7.1 (Hunter, 2007), and Numpy v1.24.3 (Harris et al., 2020) were used after the read mapping and coverage calculations were completed. Color intensity in the heatmap, varying from light to dark, represents percentage coverage. In addition to the percentage coverage values, the visualization included the depth of coverage to assist in the interpretation of the data.

## Results

All the raw reads (ONT nanopore and Illumina Miseq) are publicly available in the NCBI Sequence Read Archive (SRA): Bioproject: PRJNA944244, https://www.ncbi.nlm.nih.gov/bioproject/?term=PRJNA944244.

### Library preparation and sequencing efficiency

The aim of this study was to develop and evaluate two types of nanopore sequencing strategies, rdTotalRNA sequencing and dsRNAcD sequencing, for detecting viruses and viroids in mixed-infected grapevine samples. The pooled dsRNA library from 24 different grapevine samples was sequenced using nanopore direct-cDNA sequencing, yielding a total of 1,754,037 reads in 24 h. The 23 barcoded samples were sequenced on another flow cell which yielded 2,921,438 reads (Supplementary File S3). In addition, to compare the use of dsRNAs and total RNAs as starting materials, three of the 23 aforementioned samples (CO-9-86 J, BV-12-16 J and BIO-15-56S) were selected at random and used for total RNA extraction and direct RNA sequencing library preparation. After the rdTotalRNA library was sequenced, the 541,972 reads produced were demultiplexed and base-called (Supplementary File S3). The dsRNA-MiSeq sequencing process also yielded 194,549, 340,946, and 460,909 sequences with quality scores above Q30 from the CO-9-86 J, BV-12-16 J, and BIO-15-56S samples, respectively. For sample processing and sequencing, rdTotalRNA sequencing (29.17 h) and dsRNAcD sequencing (37.58 h) proved to be faster than Illumina dsRNA-MiSeq sequencing (88.72 h). Nanopore rdTotalRNA sequencing was 1.3 and 3 times faster than nanopore dsRNAcD sequencing and dsRNA-MiSeq sequencing, respectively (Table 2). In terms of sequencing cost, nanopore dsRNAcD sequencing was significantly cheaper (Can$103 per sample) than nanopore rdTotalRNA sequencing (Can$350 per sample) and Illumina dsRNA-MiSeq sequencing (Can$412 per sample) (Supplementary File S4).

**Table 2:** Estimated duration of extraction, library preparation, and sequencing in each RNA extraction and sequencing method.

Step	rdTotalRNA	dsRNAcD	dsRNA-MiSeq^a^
Nucleic acid extraction	0.67 h (total RNA extraction in 12 samples)	6.00 h (dsRNA extraction in 12 samples)	6.00 h (dsRNA extraction in 12 samples)
Enzyme digestion	0.50 h (DNase I)	0.50 h (DNase I and RNase T1)	0.50 h (DNase I and RNase T1)
rRNA depletion and polyadenylation	1.00 h	–	–
cDNA synthesis	1.67 h (cDNA-RNA hybrid complex)	5.25 h (Double-stranded cDNA)	5.25 h (Double-stranded cDNA)
Library preparation	1.00 h	1.50 h	
Priming and loading the flow cell	0.33 h	0.33 h	
Sequencing	24 h	24 h	72 h
Total	29.17 h	37.58 h	h

a Based on Nextera XT DNA Library Preparation Kit protocol.

### Preprocessing, clustering and error correction of long reads

Three of the 23 samples sequenced using dsRNAcD and dsRNA-MiSeq were randomly chosen for additional rdTotalRNA sequencing (the results for the remaining 20 samples are available in Zenodo: 10.5281/zenodo.7764376). The statistics on the raw data revealed that, even though all the barcoded samples were pooled together in equal concentrations, the number of sequenced reads varied between samples (Table 3). Nanopore sequencing protocols are optimized for long reads. Because several bead purification steps were carried out at different ratios during library preparation, the samples containing short nucleic acid fragments lost more sequences, which resulted in fewer reads than those containing long fragments (Oxford Nanopore Technologies, 2016). The mean quality of reads in all samples was improved by trimming and filtering (Table 3 and Supplementary File S3). In addition, removing unwanted host-related reads from the rdTotalRNA datasets revealed a 47–67% proportion of host-related raw reads, while the proportion of host-related reads in the dsRNAcD datasets ranged from 21 to 65%. The error correction and clustering of reads also increased the mean quality of corrected reads in both types of nanopore sequencing datasets before taxonomic classification (Table 3).

**Table 3:** Overview of raw data preprocessing, filtering, trimming, clustering and error correction in nanopore sequencing.

		CO-9-86 J			BV-12-16 J			BIO-15-56S		
		Raw data^a^	Trimmed & filtered^b^	Corrected^c^	Raw data	Trimmed & filtered	Corrected	Raw data	Trimmed & filtered	Corrected
rdTotalRNA^d^	Mean length (bp)	660	797.2	799.7	632.4	782.6	785.5	677.4	785.3	787.1
	Mean quality	10.8	11.5	12	10.8	11.5	12	11	11.6	12.1
	Number of reads	164,010	71,307	71,304	115,457	58,713	58,713	95,952	51,506	51,506
	N50 read length	980	1,320	1,341	986	1,314	1,338	1,027	1,305	1,326
	Total bases	108,246,484	56,847,995	57,019,794	73,014,808	45,951,225	46,119,065	64,994,220	40,446,155	40,538,263
dsRNAcD^e^	Mean length (bp)	643.1	680.5	605.4	591.5	656.4	598.4	747.6	750.2	662.6
	Mean quality	11.9	11.6	11.7	12	11.7	12	11.6	11.8	11.9
	Number of reads	42,008	31,519	31,515	49,754	16,971	16,968	84,930	67,112	67,092
	N50 read length	892	940	782	765	893	789	951	977	829
	Total bases	27,013,384	21,448,156	19,079,605	29,431,736	11,139,991	10,154,222	63,496,915	50,349,514	44,456,802

a Raw data after base-calling.

b Trimming and filtering of reads to remove low-quality bases with a high probability of being called incorrectly; host-related reads were also removed in this step.

c Read clustering and error correction.

d rdTotalRNA: rRNA-depleted total RNA datasets that were sequenced with a direct RNA sequencing kit.

e dsRNAcD: dsRNA datasets that were sequenced with a direct cDNA sequencing kit.

### Nanopore data analysis strategy 1: Centrifuge and Recentrifuge (Cent&rec)

For both dsRNAcD and rdTotalRNA sequencing datasets, a customized index database containing complete genome sequences of all possible viruses was used by the Centrifuge classifier for taxonomic classification (Kim et al., 2016). The Centrifuge classifier is a sensitive, high-speed classifier designed to classify sequences and accurately process millions of reads in a few minutes (Watts et al., 2019). The frequency of reads that could not be taxonomically assigned to subject reads in our customized index database varied between the dsRNAcD and rdTotalRNA libraries, ranging from 36.5 to 55% for the dsRNAcD libraries and from 1.5 to 16% for the rdTotalRNA libraries (Table 4). Some of the unassigned reads in the dsRNAcD libraries may have been related to novel virus species or known viruses that were not present in our database.

**Table 4:** Classification performance by Centrifuge for datasets obtained by dsRNAcD and rdTotalRNA sequencing, with the classified and the unclassified portion (%) of reads.

	CO-9-86 J	BV-12-16 J	BIO-15-56S			
	dsRNAcD	rdTotalRNA	dsRNAcD	rdTotalRNA	dsRNAcD	rdTotalRNA
Total reads	31,515	71,304	16,968	58,713	67,092	51,506
Classified reads	17,326	60,855	10,762	49,187	30,004	50,869
Classified portion	55%	85.5%	63.5%	84%	45%	98.5%
Unclassified portion	45%	14.5*%*	36.5%	16%	55%	1.5%

**Figure 3:**
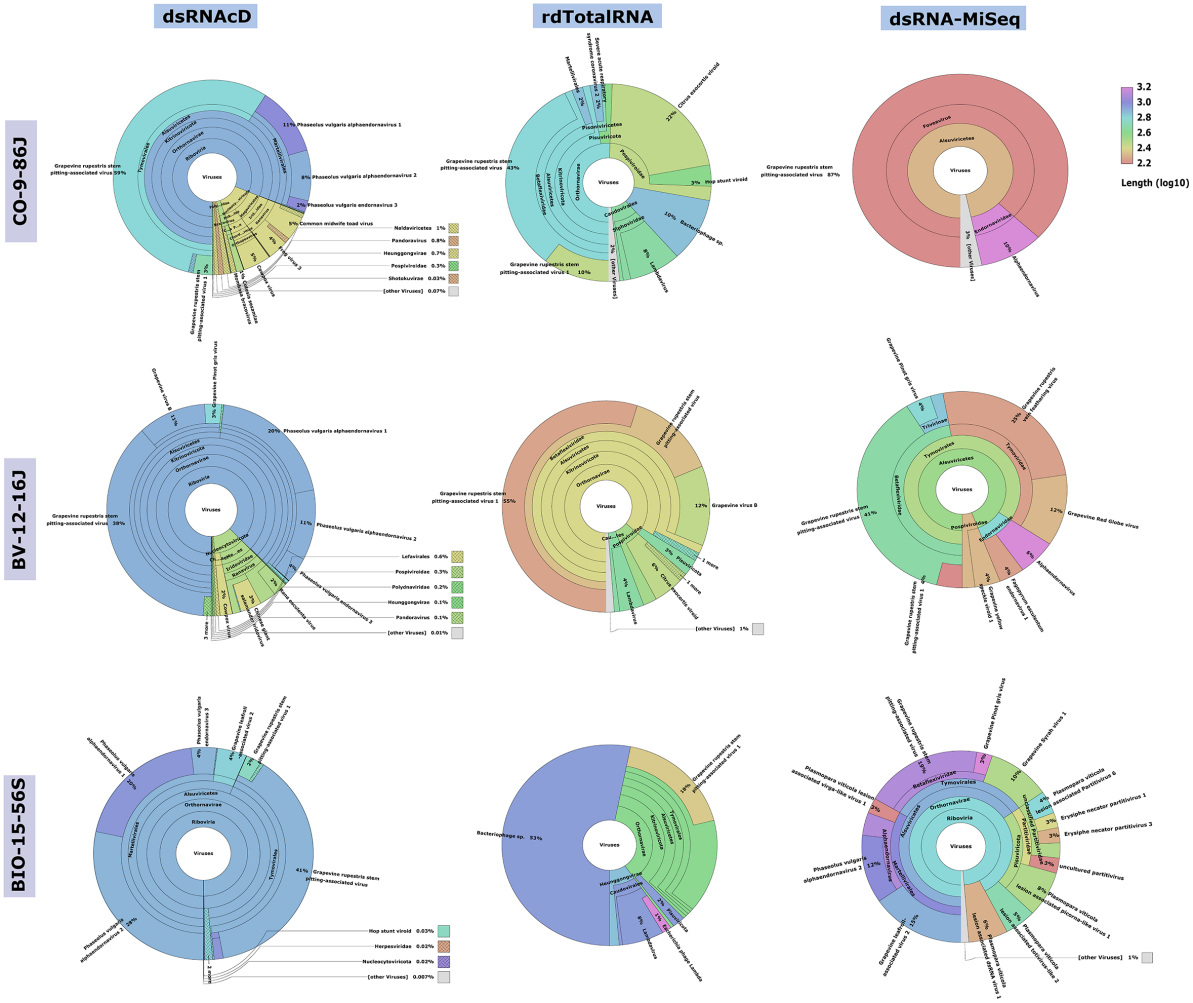
Comparative analysis by Recentrifuge software of the Centrifuge results. Screenshots of the Recentrifuge HTML interface for different samples and sequencing technologies are shown. The virus superkingdom is drawn in the center, with a hierarchical pie chart. Depending on the confidence level associated with the taxonomic classification, the background color varies for each taxon. (For the results from the other 20 samples, see available in Zenodo: 10.5281/zenodo.7764376.)

Although different sequencing platforms and analysis workflows were used for nanopore and MiSeq sequencing, we used the same index database for the taxonomic classifications of the long- and short-read datasets. The Recentrifuge software (Martí, 2019) was used to perform a comparative analysis of the results of the taxonomic classification, with both the score-based visual results and statistical results extracted in the form of HTML charts (Figure 3) and Excel files (available in Zenodo: 10.5281/zenodo.7764376). Host-related reads were initially subtracted from all the libraries to improve the accuracy of the taxonomic classification and to increase the viral read portions. Several studies haves shown that subtracting host-coextracted sequences from the short- and long-read datasets reduces the CPU time required for taxonomic classification and helps to reveal and characterize viral sequences present at low titers (Daly et al., 2015; Miller et al., 2018). The viral portion of assigned reads in the dsRNA libraries sequenced by dsRNAcD and dsRNA-MiSeq were significantly higher (in the range of 85 to 95%) than those in the rdTotalRNA libraries (in the range of 6 to 21%) (available in Zenodo: 10.5281/zenodo.7764376).

**Figure 4:**
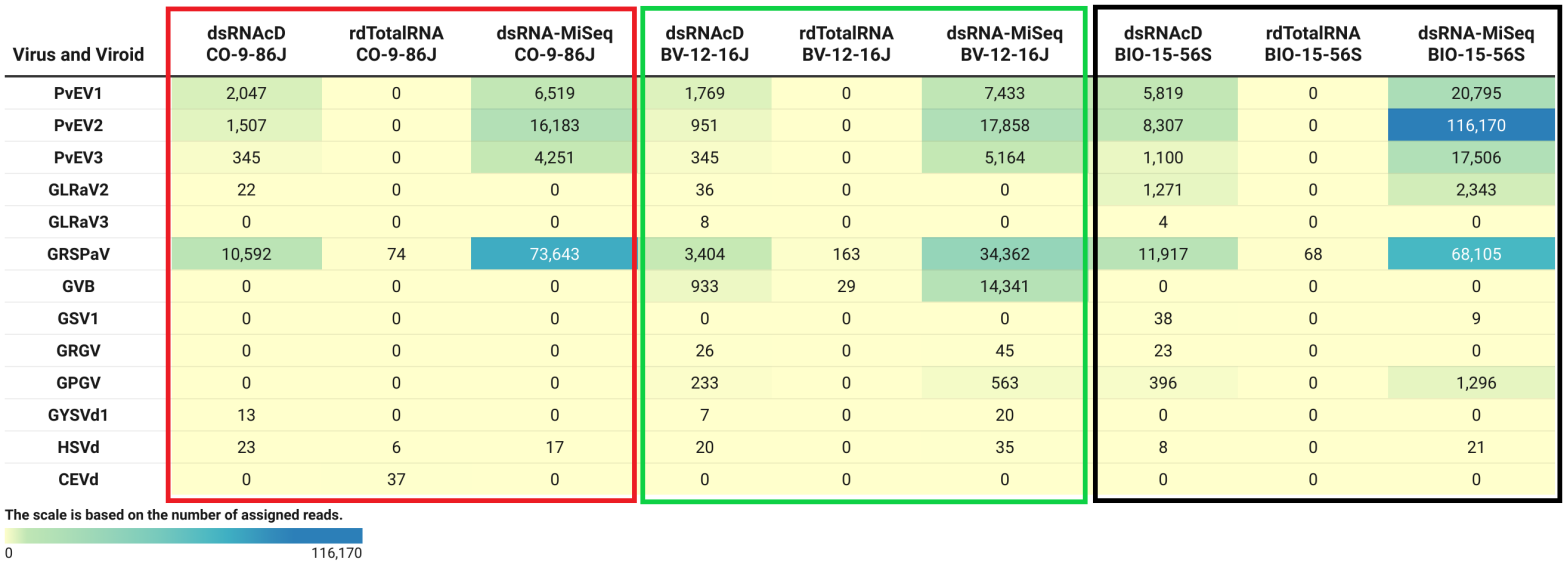
The number of assigned reads associated with grapevine viruses and viroids in different datasets. PvEV1: *Phaseolus vulgaris* endornavirus 1; PvEV2: *Phaseolus vulgaris* endornavirus 2; PvEV3: *Phaseolus vulgaris* endornavirus 3; GLRaV2: grapevine leafroll-associated virus 2; GLRaV3: grapevine leafroll-associated virus 3; GRSPaV: grapevine rupestris stem pitting-associated virus; GVB: grapevine virus B; GSV1: grapevine Syrah virus 1; GRGV: grapevine red globe virus; GPGV: grapevine pinot gris virus; GYSVd: grapevine yellow speckle viroid; GYSVd1: grapevine yellow speckle viroid 1; HSVd: hop stunt viroid; CEVd: citrus exocortis viroid. (The heat map table was generated in https://www.datawrapper.de).

The spiked positive control bean viruses (PvEV1, PvEV2 and PvEV3) were detected in all dsRNA samples. The dsRNAcD and dsRNA-MiSeq sequencing methods detected the same grapevine viruses in most of the samples. In contrast, rdTotalRNA sequencing failed to detect most of the viruses found by dsRNAcD and dsRNA-MiSeq (Table 4). However, it performed better on viruses found in large numbers (based on the number of reads) in a sample, such GRSPaV and GVB in the Betaflexiviridae family. These results suggest that nanopore dsRNAcD sequencing is more sensitive to low-abundance viruses than rdTotalRNA sequencing and produces similar results to dsRNA-MiSeq. In addition to the three samples presented in this section (Figures 3, 4), 20 other samples analyzed using dsRNA-MiSeq and dsRNAcD sequencing also provided similar results (available in Zenodo: 10.5281/zenodo.7764376), demonstrating the repeatability and accuracy of the dsRNAcD sequencing method.

The Cent&Rec workflow was also used to compare the performance of dsRNA and total RNA in viroid detection. One unexpected viroid species, citrus exocortis viroid (CEVd), was detected exclusively in the rdTotalRNA sequencing results for the CO-9-86 J sample. However, the absence of CEVd in the dsRNAcD and dsRNA-MiSeq sequencing results raises the suspicion that the reads assigned to CEVd in the rdTotalRNA dataset might be host related or from other microorganisms. To test this, the CEVd-assigned reads were extracted from the rdTotalRNA dataset from the CO-9-86 J sample; BLAST alignment against the ViroidDB and NCBI-nt databases showed that all CEVd-assigned reads were related to grapevines (*Vitis* spp.) (Supplementary File S5). Using the subject ID, the CEVd sequence (FJ751964.1) in ViroidDB that was assigned to our sequences was retrieved from the NCBI website. According to the BLAST alignment against the NCBI-nt database, a 99.46% similarity was found between the CEVd sequence in ViroidDB and the sequences of grapevines (*Vitis* spp.), which indicated that the sequence was mistakenly annotated as CEVd in the ViroidDB database. Since the number of plant-origin reads in the dsRNAcD datasets was smaller than in the rdTotalRNA datasets, this indicates that dsRNAs sequencing was more reliable than rdTotalRNA sequencing in viroid detection, even with the use of a database dedicated to and specializing in viral-like entities.

### Data analysis strategy 2: DIAMOND and MEGAN (DIA&MEG)

**Figure 5:**
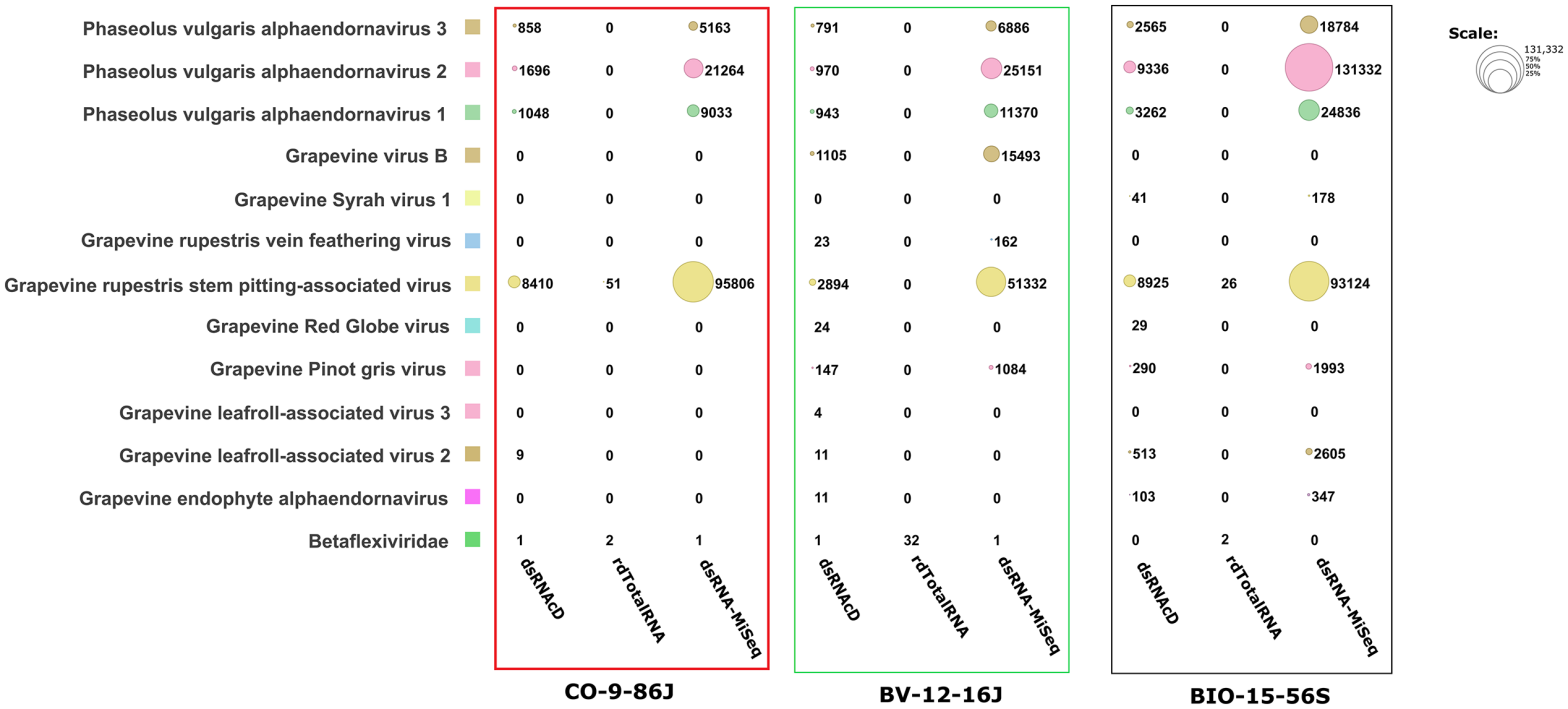
Comparative visualization of DIAMOND results by MEGAN software. Multiple datasets (1. Nanopore dsRNAcD sequencing; 2. Nanopore rdTotalRNA sequencing; and 3. dsRNA-MiSeq sequencing) were uploaded to MEGAN and the relative abundance of reads in each sample was extracted.

In addition to the Cent&Rec workflow, we also used the DIA&MEG workflow for virus detection. DIAMOND was used to align the sequenced reads against the protein database (NCBI-nr), and then MEGAN binned the alignments based on taxonomic and functional information. Because viroid genomes do not encode proteins, this strategy can only be used to detect viruses. The viruses detected by Cent&Rec in the dsRNAcD and dsRNA-MiSeq datasets were also detected by DIA&MEG (Figure 5). Besides the results presented in Figure 5, we also analyzed 20 different datasets of dsRNA samples that were sequenced by dsRNAcD and dsRNA-MiSeq to verify the accuracy of the Cent&Rec workflow compared with the DIA&MEG workflow (available in Zenodo: 10.5281/zenodo.7764376).

**Figure 6:**
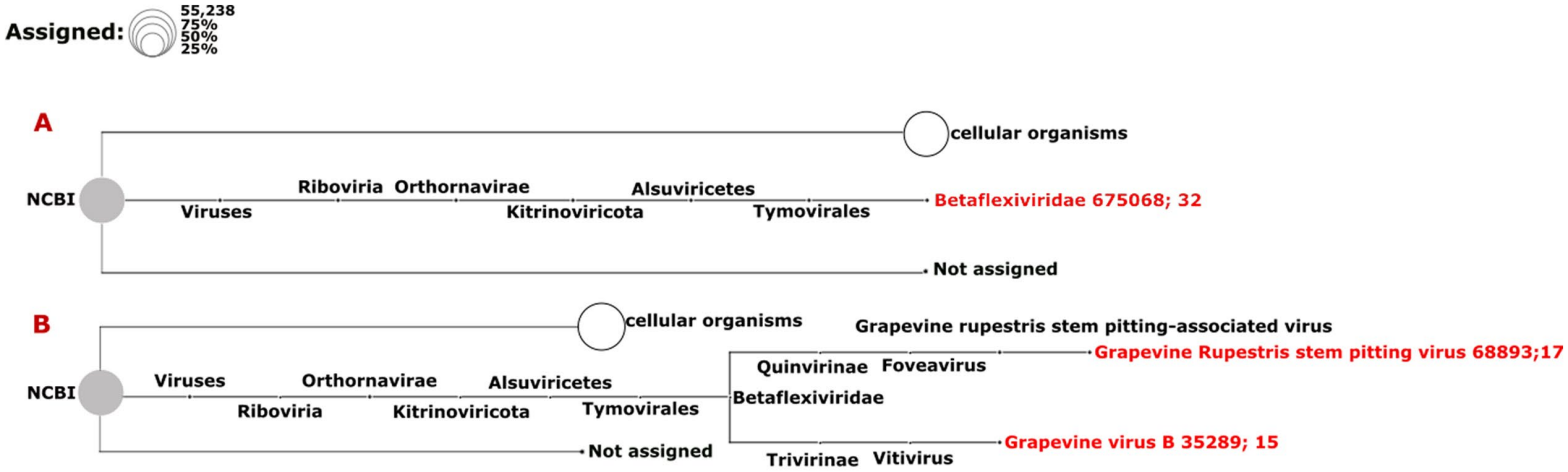
Assignment of viral reads to taxa using different thresholds in the rdTotalRNA dataset from the BV-12-16 J sample. **(A)** Taxonomic binning was done using the default parameters in the meganization option of the MEGAN software. **(B)** Two thresholds, “Min Support Percent” and “Min Support,” were turned off (=0) when meganization was performed. The two numbers following the virus name (red color) consist of the NCBI taxonomy ID followed by a number that indicates the number of assigned reads.

The results from the DIA&MEG workflow were generally similar to those from the Cent&Rec workflow except for the detection of GRSPaV, GVB and grapevine endophyte alphaendornavirus (GEEV). GRSPaV was detected in only two of the three rdTotalRNA datasets when using the DIA&MEG workflow with default parameters, but was detected in all three rdTotalRNA datasets when using the Cent&Rec workflow (Figure 5). In the rdTotalRNA dataset from the BV-12-16 J sample, GVBv and GRSPaV (from the family Betaflexiviridae) were detected by Cent&Rec, but not by DIA&MEG. However, 32 reads in the rdTotalRNA dataset from the BV-12-16 J sample were taxonomically assigned at the family level to Betaflexiviridae (Figure 6A). This result can be explained by the default threshold options in the MEGAN software. However, when the threshold options “Min Support Percent” and “Min Support” were turned off (=0), these 32 reads were taxonomically assigned at the species level to GRSPaV and GVB (Figure 6B). In addition, the rdTotalRNA dataset from the BV-12-16 J sample had very few viral reads (34 reads), even after the host reads were removed. The small number of viral reads in this dataset makes accurate taxonomic classification risky. In contrast, both the GRSPaV and GVB viruses in the dsRNAcD and dsRNA-MiSeq datasets were binned correctly based on their taxonomy, even when using the default threshold options in the MEGAN software.

A difference was also observed in the detection of GEEV in the dsRNA and total RNA datasets. No reads in the rdTotalRNA dataset from the BIO-15-56S sample were mapped to the GEEV genome, while the dsRNAcD and dsRNA-MiSeq datasets did provide reads mapped to the virus genome (Figure 5). In addition, the Cent&Rec workflow did not allow GEEV to be detected in the BIO-15-56S sample, suggesting that the results obtained with this workflow should be viewed with caution. Since GEEV was detected exclusively using the DIA&MEG workflow, BLAST annotation was performed against the NCBI-nt and NCBI-nr databases using BlastN and BlastX to ensure that the reads assigned to GEEV were not false positives. Only Blastx results confirmed the presence of GEEV in the BIO-15-56S sample, corresponding to the results obtained with DIA&MEG (Supplementary File S6).

### Viral genome coverage

**Figure 7:**
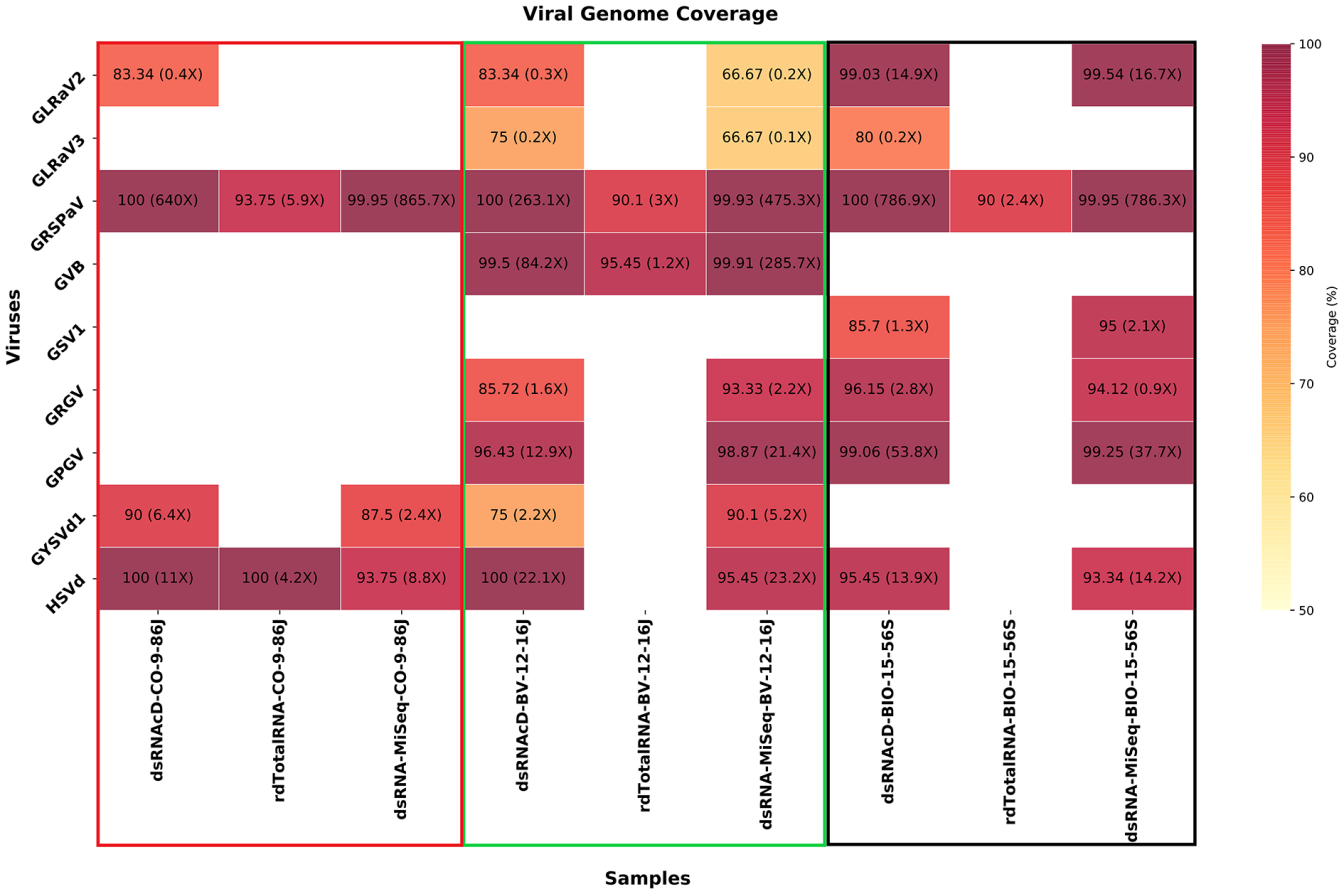
A heatmap illustrating the percentage of viral genome coverage and depth for three different grapevine samples. In each cell, the associated number represents the percentage of genome coverage, followed by the depth of coverage in parentheses. Colors that are darker indicate higher genome coverage percentages.

To verify taxonomy classification results, viral genome coverage was also examined. We observed a wide range of coverage and depth values among the various detected viruses across various samples. GLRaV2 was detected in CO-9-86 J, BV-12-16 J, and BIO-15-56S samples through dsRNAcD sequencing, with genome coverage of 83.34, 83.34, and 99.03%, respectively (Figure 7). However, the coverage depth was comparatively low in CO-9-86 J and BV-12-16 J samples, with a maximum of 0.4X and 0.3X, respectively. GLRaV2 was not detected in all samples through rdTotalRNA sequencing, matching the taxonomy classification results. In all samples, GRSPaV was detected through all sequencing platforms, reaching full or near full genome coverage (ranging between 90–100%). For GRSPaV, the depth of coverage varied widely, from 2.4X in rdTotalRNA-BIO-15-56S to 865.7X in dsRNA-MiSeq-CO-9-86 J. In the BV-12-16 J sample, grapevine virus B (GVB) showed genome coverage of 99.5% in dsRNAcD and 99.91% in dsRNA-MiSeq, with depths of coverage of 84.2X and 285.7X, respectively (Figure 7). It had a high coverage percentage in the rdTotalRNA dataset, but a much lower coverage depth than dsRNA libraries. GSV1 was detected by dsRNAcD with 85.7% genome coverage and 1.3X depth as well as by dsRNA-MiSeq with 95% genome coverage and 2.1X depth, however, rdTotalRNA did not detect this virus. HSVd was detected in all samples except in rdTotalRNA of BV-12-16 J and rdTotalRNA of BIO-15-56S, with genome coverage ranging from 93.34 to 100% and depth of coverage ranging from 4.2X to 23.2X. There were also a number of other viruses and viroids detected in some samples, including GLRaV3, GRGV, GPGV, and GYSVd1, all showing varying levels of genome coverage and depth, which represents a complex viral communities present in our samples (Figure 7).

## Discussion

The aim of this study was to develop and evaluate two nanopore sequencing strategies for detecting viruses and viroids in mixed-infected grapevine samples: direct-RNA sequencing and direct-cDNA sequencing. In the study, we tried to address two main questions on grapevine virus and viroid diagnostics. First, which starting material is better—total RNA or dsRNA—in single-molecule nanopore sequencing for grapevine virus and viroid detection? Second, can a nanopore-based diagnostic tool be cost effective in relation to conventional MiSeq sequencing in grapevine virus and viroid detection and to what extent? In this study, the viral reads were short (approximately 800 bp on average) and not sufficiently abundant for *de novo* assembly. Low coverage, quality, and quantity of dsRNA sequencing libraries can cause challenges for *de novo* assemblers to assemble viral genome (Amoia et al., 2022). *De novo* assemblers are primarily designed for reconstructing microbial and eukaryotic genomes and may not be the best option for assembling viral genomes using dsRNA sequencing libraries. Therefore, two different bioinformatics workflows based on read classification by Rattle and Cent&Rec and DIA&MEG taxonomic classification, were used in the detection of grapevine viruses and viroids.

Until now, nanopore dsRNA sequencing has been used to identify or characterize viruses in single virus infections (Amoia et al., 2022; Dong et al., 2022). Total RNA extraction is the common method used in the published literature for identifying and characterizing the virome using nanopore sequencing technology. Several limitations in using dsRNA for viral diagnostics have slowed advances in its use, including the labor-intensive extraction methods required and the notion that dsRNA may not be produced by some viruses. In this study, the preparation of a dsRNAcD sequencing library was found to take more time than that of a rdTotalRNA sequencing library. However, dsRNAcD sequencing allowed more samples to be processed simultaneously on one flow cell (23 samples), and the sequencing cost per sample was three times lower (Can$103) than that of rdTotalRNA sequencing (Can$350). The library preparation protocol for rdTotalRNA sequencing requires two additional enzymatic steps (rRNA-depletion and polyadenylation) in order to remove the unwanted rRNAs from samples and to capture non-poly(A)-tailed viruses, which increases the sequencing cost per sample. However, when dsRNA is used as the starting material and random primers are employed for cDNA synthesis, these enzymatic steps can be eliminated, resulting in a much lower number of unwanted sequences in dsRNAcD datasets than in rdTotalRNA datasets. It remains to be determined how efficient these sequencing protocols are in terms of the yield of viral reads and the accuracy of virus identification, as well as in the ease of bioinformatics data analysis.

The viral read proportions in the dsRNA datasets were significantly higher than those in the rdTotalRNA datasets. For example, in the dsRNAcD dataset from the CO-9-86 J sample, 89% of assigned reads were virus related while, in the rdTotalRNA dataset, only 6% of assigned reads were virus related. Not only did rdTotalRNA sequencing fail to detect all the viruses present in our samples, but three other issues arose with it during data analysis. First, plant-derived reads were incorrectly assigned to the Citrus exocortis viroid (CEVd) in the CO-9-86 J sample. This issue had also been reported in a previous study, where a plant sequence was misannotated as CEVd (Lelwala et al., 2022). However, we did not observe this misannotation in the dsRNAcD and dsRNA-MiSeq datasets from the same sample. By using dsRNA as a starting material, plant host-related sequences, including those that are misannotated as viroids in the database, were effectively excluded. This resulted in a smaller proportion of plant-derived sequences in the dsRNA datasets, thereby reducing the likelihood of encountering false positives due to misannotations. Consequently, using dsRNA for detecting viruses and viroids through taxonomic classification increases the reliability of the results and decreases the likelihood of false positive outcomes. Second, a problem arose in the taxonomic classification of viruses in the rdTotalRNA dataset from the BV-12-16 J sample. Since the abundance of GRSPaV and GVB reads in the rdTotalRNA dataset was low, taxonomic classification at the species level was not possible when using the MEGAN default settings. The taxonomic classification of long reads, specifically viral reads, can be affected by a number of factors, including the viral load and mutation rate. Indeed, species with low abundance may be discarded depending on the threshold options selected in the taxonomic classification software. In order to apply thresholds effectively and avoid discarding low abundance species, it is important to take into consideration the type of study, the read depth, and the classifier software used (McIntyre et al., 2017). Furthermore, most taxonomic classifiers are designed to classify bacteria or microorganisms, and not viruses. Viruses’ high mutation rate must be taken into account, and consequently the software’s mismatch threshold may need to be adjusted (Breitwieser et al., 2017). In contrast, both dsRNAcD and dsRNA-MiSeq sequencing yielded enough viral read counts for the accurate detection of both GRSPaV and GVB when using the default MEGAN thresholds.

Third, the detection of GEEV was also affected by the type of starting materials used, as well as the bioinformatics workflow. The dsRNAcD and dsRNA-MiSeq datasets from one sample contained GEEV-related viral reads, while the rdTotalRNA dataset from the same sample did not. In addition, the Cent&Rec workflow was not able to detect GEEV in all the datasets from same sample. In the dsRNA datasets, although the proportion of viral-assigned reads was high, a substantial number of unassigned reads also occurred. While reverse transcription artifacts could explain the high number of unassigned reads (Cebriá-Mendoza et al., 2021) in the dsRNA sequencing datasets, some of these unassigned reads may have been related to novel virus strains or species. Consequently, these unassigned reads could be characterized using hybrid assembly and comparative analysis. Through BLASTn and BLASTx, we verified that the detected GEEV was not a false positive. According to our findings, the sequence similarity with the NCBI record (YP_007003829.1) was observed at the protein level. Therefore, grapevine samples may harbor different strains or haplotypes of GEEV, indicating GEEV is highly diverse genetically. As a result, there is a need for comprehensive research focused on genetic diversity within GEEV and the possibility of discovering a novel endornavirus. This current manuscript does not cover this potentially intriguing discovery, although we have been conducting more lab experiments to test these hypotheses (not showed). According to Al Rwahnih et al. (2009), dsRNA sequencing provides a greater number of unique cDNA sequences than total RNA sequencing and consequently, dsRNA sequencing has a greater potential in the discovery of new viruses. Indeed, several novel grapevine viruses, including grapevine Syrah virus-1, grapevine red blotch virus, and others, were discovered through dsRNA sequencing (Al Rwahnih et al., 2009; Sudarshana et al., 2015). Therefore, the use of dsRNA not only allows known viruses and viroids to be detected, but increases the potential for discovering new plant and non-plant virus species.

The comparative efficiency of different sequencing technologies and approaches for detecting grapevine viruses and viroids was further evaluated by analyzing viral genome coverage and depth. Genome coverage results of taxonomy classification methods, DIA&MEG and Cent&Rec, were in similar. In the tested samples, rdTotalRNA was not able to detect all viruses and viruses. The reason for this might be that total RNA contains a vast majority of host transcripts, which increases the complexity of the data and potentially overshadow low-titer viral reads. Despite the fact that the rdTotalRNA sequencing can provide a broad overview of both viral and host transcripts, it appears to be less effective for detecting viruses, especially those that are present at lower titers (Pecman et al., 2022). In contrast, double-stranded RNA (dsRNA), sequenced by dsRNAcD and dsRNA-MiSeq, proved more effective at detecting various viruses. Because dsRNA acts as an intermediate product during the viral replication process or as an erroneous product resulting from the bidirectional transcriptional, it reducing noise and enhancing the likelihood of detecting viral reads (Decker et al., 2019). This could explain the differences in genome coverage and depth observed in the detection of viruses among the different extraction methods (dsRNA, total RNA). In spite of the low depth of coverage, certain viruses were still detectable such as GSV1 and GRGV, illustrating the sensitivity of the sequencing technologies used, dsRNAcD and dsRNA-MiSeq. Furthermore, the substantial variability in depth of coverage observed within and between samples, even with full or near-full genome coverage, illustrates the dynamic nature of viral titers within hosts.

The remaining question is how cost-effective nanopore dsRNA sequencing is in relation to Illumina MiSeq sequencing? To determine which sequencing technology is the most appropriate for diagnostic laboratory work and disease management operations, we compared the cost-effectiveness of MiSeq and nanopore sequencing in terms of library preparation time, ease of use, sequencing cost, and data analysis requirements. In general, Illumina sequencing is not cost effective at a small scale, discouraging the use of this technology in day-to-day diagnostic activities in diagnostic labs. Indeed, the cost of the sequencer and the minimum number of samples required (50 to 60 samples) to provide cost-effectiveness make Illumina sequencing less suitable for small diagnostic labs, and nanopore sequencing is a viable alternative to MiSeq sequencing in this situation. In our study, the estimated cost of nanopore dsRNA sequencing was Can$103 per sample, while Gaafar and Ziebell (2020) estimated the cost of virus detection using dsRNA-MiSeq sequencing to be Can$412 per sample, suggesting that nanopore dsRNA sequencing is one fourth the cost of dsRNA-MiSeq sequencing. In addition, a nanopore dsRNAcD sequencing library can be prepared and sequenced in 37.58 h, while a dsRNA-MiSeq library takes nearly 88.75 h to prepare and sequence (Table 2). Our analysis showed that the list of detected viruses obtained with nanopore dsRNAcD sequencing using both proposed data analysis workflows (DIA&MEG and Cent&Rec) was similar to the dsRNA-MiSeq results in all samples. Although choosing a pipeline to analyze MiSeq data did not present any difficulties, some challenges arose in analyzing the nanopore sequencing data. Similar to the situation described by Amoia et al. (2022), our dsRNAcD libraries provided viral reads in insufficient numbers and of inadequate quality to meet the requirements of long-read-based de-novo assembler software. Therefore, the software was not able to assemble large numbers of long viral contigs. Most de-novo assemblers are designed for reconstructing complete microbial and near-complete eukaryotic genomes. Therefore, taxonomic classification tools had to be used to analyze our raw data. In addition, because raw data trimming, filtering, and error correction take more time in nanopore sequencing, virus detection was slower than in MiSeq data analysis. We performed an additional data preprocessing step that involved the subtraction of host-origin reads from the trimmed and error-corrected datasets, with the goal of increasing viral read counts and improving the taxonomic classification of viruses. Although the DIA&MEG workflow generally takes a long time to run when analyzing large datasets, such as environmental metagenomic datasets (Buchfink et al., 2021; Gautam et al., 2022), it only took a short time (around 2 h) with our datasets. This workflow provides the user with a wide range of options, including displaying a taxonomic tree of the detected viruses, extracting functional information from assigned reads, comparing different samples, and analyzing and comparing short-read and long-read sequencing datasets. However, the Cent&Rec workflow was faster than DIA&MEG in terms of running time (around 20 to 30 min), and this workflow was able to detect viroids, unlike DIA&MEG. Our study demonstrated that dsRNAcD sequencing can effectively compete with MiSeq sequencing in detecting viruses and viroids.

## Conclusion

The purpose of this study was to examine the ability of nanopore direct cDNA and direct RNA sequencing to simultaneously detect grapevine viruses compared to that of Illumina MiSeq sequencing. According to our results, dsRNA is a more reliable starting material for library preparation than total RNA in terms of identifying grapevine viruses and viroids. The dsRNA sequencing results for all samples were similar to those from dsRNA-MiSeq sequencing. In addition, the rRNA depletion step did not improve grapevine virus detection in the total RNA libraries despite the increase in the cost per sample. In contrast, when dsRNA was sequenced using a direct cDNA sequencing kit, not only were more samples (23 samples) multiplexed and sequenced simultaneously on one flow cell, but also the total cost of sequencing fell significantly. However, the current dsRNA purification protocol needs to be improved and optimized in order to increase the efficiency of time and effort. In conclusion, the study demonstrated that dsRNAcD sequencing can be an affordable routine diagnostic tool for detecting plant viruses.

## Data availability statement

The datasets presented in this study can be found in online repositories. The names of the repository/repositories and accession number(s) can be found in the article/Supplementary material.

## Author contributions

MF, VJ, and PM conceived and designed the research. VJ and PL performed the experiments. AP, VJ, and MF conceived the bioinformatic pipelines. VJ made the graphical representation. DS-C and PL provided technical assistance. VJ and MF drafted the manuscript. PM, AP, PL, and DS-C edited, corrected and improved the manuscript. All authors contributed to the article and approved the submitted version.

## Funding

This study was supported by grants from the Ministère de l’Agriculture, des Pêcheries et de l’Alimentation du Québec under the Cellule d’innova tion des méthodologies de diagnostic des ennemis des cultures (CIMDEC) initiative and Agriculture and Agri-Food Canada under the Collaborative Framework priority (J-002411).

## Conflict of interest

The authors declare that the research was conducted in the absence of any commercial or financial relationships that could be construed as a potential conflict of interest.

## Publisher’s note

All claims expressed in this article are solely those of the authors and do not necessarily represent those of their affiliated organizations, or those of the publisher, the editors and the reviewers. Any product that may be evaluated in this article, or claim that may be made by its manufacturer, is not guaranteed or endorsed by the publisher.

## Footnotes

^1^https://www.protocols.io/view/double-stranded-rna-extraction-by-cellulose-4r3l2odrjv1y/v1

^2^https://www.protocols.io/view/synthesis-of-double-strand-cdna-ds-cdna-from-viral-bp2l69nddlqe/v1
